# *GST* null polymorphisms may affect the risk of coronary artery disease: evidence from a meta-analysis

**DOI:** 10.1186/s12959-020-00234-x

**Published:** 2020-09-01

**Authors:** Hongling Su, Yunshan Cao, Jing Li, Yan Zhu, Xuming Ma

**Affiliations:** Department of Cardiology, Gansu Provincial People’s Hospital, No. 204 of Donggang West Road, Lanzhou, 730000 Gansu China

**Keywords:** Glutathione S-transferase (*GST*), Null polymorphisms, Coronary artery disease (CAD), Meta-analysis

## Abstract

**Background:**

Whether glutathione S-transferase (*GST*) null polymorphisms, namely *GSTM1* null, *GSTP1* null and *GSTT1* null polymorphisms, influence the risk of coronary artery disease (CAD) or not remains unclear. Thus, the authors performed a meta-analysis to more robustly estimate associations between *GST* null polymorphisms and the risk of CAD by integrating the results of previous publications.

**Methods:**

Medline, Embase, Wanfang, VIP and CNKI were searched comprehensively for eligible studies, and 45 genetic association studies were finally selected to be included in this meta-analysis.

**Results:**

We found that *GSTM1* null polymorphism was significantly associated with the risk of CAD in overall population (OR = 1.37, *p* = 0.003) and mixed population (OR = 1.61, *p* = 0.004), *GSTP1* null polymorphism was significantly associated with the risk of CAD in overall population (OR = 1.23, *p* = 0.03), whereas *GSTT1* null polymorphism was significantly associated with the risk of CAD in overall population (OR = 1.23, *p* = 0.02), Caucasians (OR = 1.23, *p* = 0.02) and East Asians (OR = 1.38, *p* < 0.0001).

**Conclusions:**

This meta-analysis demonstrated that *GSTM1* null, *GSTP1* null and *GSTT1* null polymorphisms were all significantly associated with an increased risk of CAD.

## Background

Coronary artery disease (CAD) is featured by stenosis or even occlusion of coronary arteries, and their associated myocardial ischemia or infarction [1, 2]. The exact cause and pathogenesis of CAD are still nuclear despite extensive researches. Nevertheless, accumulating evidence supports that genetic factors play a crucial part in its development. First, family aggregation of CAD has been observed extensively, and past twin studies have demonstrated that the heredity grade of CHD can be as high as 50% [3, 4]. Second, numerous genetic polymorphisms have been found to be associated with an increased risk of CAD by previous genetic association studies, and screening of common causal mutations has also been demonstrated to be an efficient way to predict the individual risk of developing CAD [5, 6]. Overall, these findings jointly indicate that genetic architecture is important for the occurrence and development of CAD.

Oxidative stress, characterized by accumulation of free radicals, membrane lipid peroxidation and DNA damage, has been found to play a critical role in the pathogenesis of various atherothrombotic disorders including CAD [7, 8]. Glutathione-S-transferases (GSTs) are a group of enzymes that play vital roles in regulating cellular detoxification of various exogenous toxins [9]. Moreover, it has been shown that GSTs have anti-oxidation effects and they can protect cells against oxidative stress and its associated DNA damage [10]. Previous experimental studies have demonstrated that *GST* null polymorphisms, which include null polymorphisms of *GSTM1* (mu), *GSTP1* (pi) and *GSTT1* (theta) can result in a diminished gene expression level and a reduced enzymatic activity of GST [11, 12]. Consequently, it is biologically plausible that *GST* null polymorphisms may also affect the risk of CAD. Over the last decade, investigators across the world have repeatedly attempted to assess the associations between *GST* null polymorphisms and the risk of CAD, with inconsistent findings. So a meta-analysis was performed by us to more robustly estimate the associations between *GST* null polymorphisms and the risk of CAD by integrating the results of previous publications.

## Methods

This meta-analysis was conducted in accordance with the PRISMA guideline [13].

### Literature search and inclusion criteria

Medline, Embase, Wanfang, VIP and CNKI were comprehensively searched by the authors using the below keywords: (glutathione S-transferase OR GST) AND (polymorphism OR polymorphic OR variation OR variant OR mutant OR mutation OR SNP OR genotypic OR genotype OR allelic OR allele) AND (coronary atherosclerotic heart disease OR coronary heart disease OR coronary artery disease OR ischemic heart disease OR angina pectoris OR acute coronary syndrome OR myocardial infarction OR CHD OR CAD OR IHD OR ACS OR MI). Moreover, we also manually screened the references of retrieved publications to make up for the potential incompleteness of literature searching from electronic databases.

Selection criteria of this meta-analysis were listed below: 1. Studies of case-control or cohort design; 2. Give genotypic frequencies of *GST* null polymorphisms in cases with CAD and population-based controls; 3. The full manuscript with detailed genotypic frequencies of *GST* null polymorphisms is retrievable or buyable. Articles would be excluded if one of the following three criteria is satisfied: 1. Studies without complete genotypic data of *GST* null polymorphisms in cases with CAD and population-based controls; 2. Narrative or systematic reviews, meta-analysis or comments; 3. Case series of subjects with CAD only. If duplicate reports are retrieved, we would only include the most complete one for integrated analyses.

### Data extraction and quality assessment

The authors extracted the following data items from eligible studies: 1. Last name of the leading author; 2. Year of publication; 3. Country and ethnicity of study population; 4. The number of cases with CAD and population-based controls; 5. Genotypic frequencies of *GST* null polymorphisms in cases with CAD and population-based controls. The quality of eligible publications was assessed by the Newcastle-Ottawa scale (NOS) [14], and these with a score of 7 - 9 were considered to be of good quality. Two authors extracted data and assessed quality of eligible literatures in parallel. A thorough discussion until a consensus is reached would be endorsed in case of any discrepancy between two authors.

### Statistical analyses

All statistical analyses in this meta-analysis were performed with the Cochrane Review Manager software. Associations between *GST* null polymorphisms and the risk of CAD were explored by using odds ratio and its 95% confidence interval. The statistically significant *p* value was set at 0.05. The authors used I^2^ statistics to estimate heterogeneities among included studies. The authors would use DerSimonian-Laird method, which is also known as the random effect model, to integrate the results of eligible studies if I^2^ is larger than 50%. Otherwise, the authors would use Mantel-Haenszel method, which is also known as the fixed effect model, to integrate the results of eligible studies. Meanwhile, the authors also conduct subgroup analyses by ethnic groups. The overall population (with all study subjects of eligible studies for each polymorphism included) can be divided into Caucasians, Asians or the mixed populations. If the authors specify the ethnic origin of study subjects in their publications, then we would use these data to divide the publications into different subgroups. But if the authors failed to specify the ethnic origin of study subjects in their publications, then we would use the location of the authors’ affiliations to divide the publications into different subgroups. For the mixed population, since the authors failed to specify the ethnic origin of study subjects and we could not judge the ethnic origin of study subjects from authors’ affiliations neither, it may have several scenarios, which can be a mixture of Caucasians and Africans, a mixture of Caucasians and Asians, a mixture of Africans and Asians, or a mixture of Caucasians, Asians and Africans. Stabilities of integrated results were tested by deleting one study each time, and then integrating the results of the rest of eligible studies. Publication biases were evaluated by assessing symmetry of funnel plots.

## Results

### Characteristics of included studies

**Fig. 1:**
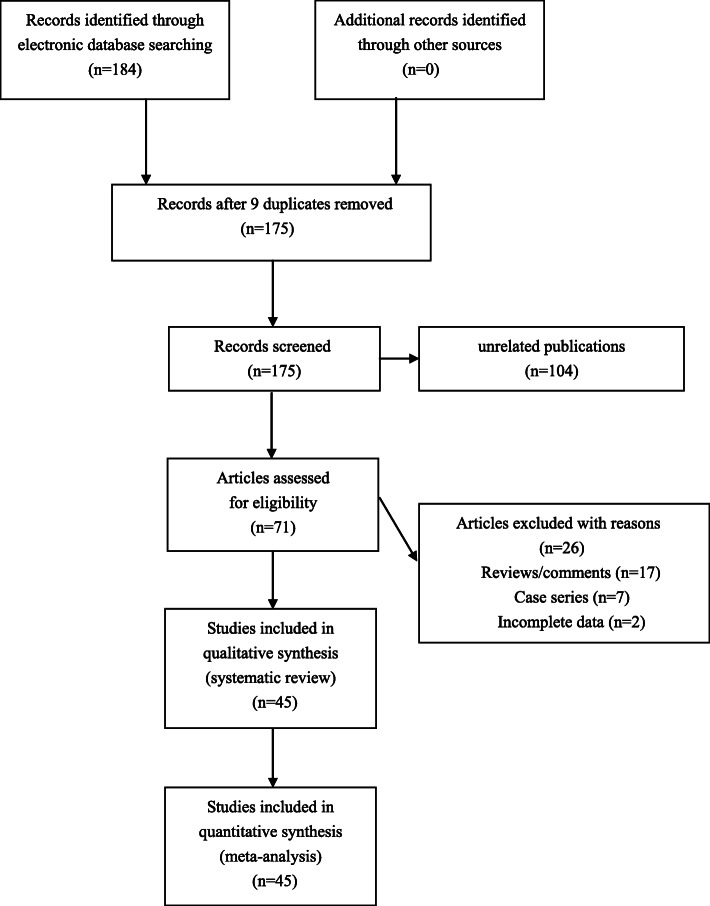
Flowchart of study selection for this meta-analysis

One hundred and eighty-four publications were retrieved by using our searching strategy. Among these publications, nine duplicate reports as well as one hundred and four unrelated publications (papers that were not about *GST* null polymorphisms and the risk of CAD) were omitted, and 71 publications were then selected to screen for eligibility. Seventeen reviews and seven case series were further excluded, and another two publications without complete genotypic data were further excluded by the authors. Totally 45 studies met the inclusion criteria, and were finally enrolled for integrated analyses (Fig. 1). The eligible studies were published between 1996 and 2020. Data extracted from eligible studies were summarized in Table 1.

**Table 1:** The characteristics of included studies in this meta-analysis

First author, year	Country	Ethnicity	Type of disease	Sample size Case/Control	Null genotype [n(%)]		NOS score
					Cases Controls		
***GSTM1 null***							
Abu-Amero 2006	Saudi Arabia	Mixed	Coronary artery disease (CAD)	1054/762	655 (62.1%)	117 (15.3%)	7
Bazo 2011	Brazil	Mixed	Coronary artery disease (CAD)	297/96	160 (53.8%)	40 (41.7%)	7
Bhat 2016	India	Mixed	Coronary artery disease (CAD)	200/200	62 (31.0%)	36 (18.0%)	8
Bhatti 2018	India	Mixed	Coronary artery disease (CAD)	562/564	217 (38.6%)	127 (22.5%)	7
Cora 2013	Turkey	Caucasian	Myocardial infarction (MI)	324/296	182 (56.1%)	143 (48.3%)	8
Cornelis 2007	Canada	Caucasian	Myocardial infarction (MI)	2042/2042	980 (48.0%)	1041 (51.0%)	7
Evans 1996	Saudi Arabia	Mixed	Coronary artery disease (CAD)	90/884	57 (63.3%)	484 (54.8%)	7
Girisha 2004	India	Mixed	Coronary artery disease (CAD)	197/198	46 (23.4%)	41 (20.7%)	7
Hayek 2006	Israel	Mixed	Coronary artery disease (CAD)	193/2399	88 (45.6%)	1142 (47.6%)	8
Kadıoğlu 2016	Turkey	Caucasian	Coronary artery disease (CAD)	29/30	17 (58.6%)	14 (46.7%)	7
Kariž 2012	Slovenia	Caucasian	Myocardial infarction (MI)	206/257	142 (69.0%)	166 (64.6%)	7
Kim 2008	Korea	East Asian	Coronary artery disease (CAD)	356/336	198 (55.6%)	191 (56.8%)	7
Li 2000	USA	Mixed	Coronary artery disease (CAD)	400/790	178 (44.5%)	354 (44.8%)	7
Macie 2009	Brazil	Mixed	Coronary artery disease (CAD)	869/1573	557 (64.1%)	789 (50.2%)	7
Manfredi 2007	Italy	Caucasian	Coronary artery disease (CAD)	169/53	99 (58.6%)	24 (45.3%)	7
Manfredi 2009	Italy	Caucasian	Coronary artery disease (CAD)	184/47	108 (58.7%)	18 (38.3%)	7
Martin 2009	USA	Mixed	Coronary artery disease (CAD)	67/63	41 (61.2%)	19 (30.2%)	7
Masetti 2003	Italy	Caucasian	Coronary artery disease (CAD)	308/122	163 (52.9%)	66 (54.1%)	8
Mir 2016	India	Mixed	Coronary artery disease (CAD)	100/100	42 (42.0%)	26 (26.0%)	8
Nomani 2011	Iran	Mixed	Coronary artery disease (CAD)	209/108	100 (47.8%)	57 (52.8%)	8
Norskov 2011	Denmark	Caucasian	Coronary artery disease (CAD)	4930/21684	2052 (41.6%)	11,362 (52.4%)	7
Olshan 2003	USA	Mixed	Coronary artery disease (CAD)	526/868	252 (47.9%)	352 (40.6%)	8
Pašalić 2017	Croatia	Caucasian	Coronary artery disease (CAD)	71/174	29 (40.8%)	69 (39.7%)	7
Phulukdaree 2012	India	Mixed	Coronary artery disease (CAD)	102/100	37 (36.3%)	18 (18.0%)	7
Pourkeramati 2020	Iran	Mixed	Coronary artery disease (CAD)	244/281	128 (52.5%)	138 (49.1%)	8
Ramprasath 2011	India	Mixed	Coronary artery disease (CAD)	290/270	128 (44.1%)	56 (20.7%)	7
Salama 2002	USA	Mixed	Coronary artery disease (CAD)	130/90	45 (34.6%)	33 (36.7%)	7
Singh 2011	India	Mixed	Myocardial infarction (MI)	230/300	56 (24.3%)	65 (21.7%)	8
Tamer 2004	Turkey	Caucasian	Coronary artery disease (CAD)	148/247	67 (45.3%)	103 (41.7%)	7
Tang 2009	China	East Asian	Coronary artery disease (CAD)	277/277	89 (32,.1%)	59 (21.3%)	7
Taspinar 2012	Turkey	Caucasian	Coronary artery disease (CAD)	122/142	51 (41.8%)	66 (46.5%)	7
Wang 2002	Australia	Caucasian	Coronary artery disease (CAD)	612/256	343 (56.0%)	153 (59.8%)	7
Wang 2008	China	East Asian	Coronary artery disease (CAD)	277/277	89 (32.1%)	59 (21.3%)	8
Wilson 2000	UK	Caucasian	Myocardial infarction (MI)	356/187	191 (53.7%)	107 (57.2%)	8
Wilson 2003	UK	Mixed	Coronary artery disease (CAD)	170/203	70 (41.2%)	107 (52.7%)	7
Yeh 2013	Taiwan	East Asian	Coronary artery disease (CAD)	458/209	253 (55.2%)	121 (57.9%)	8
Zhang 2011	China	East Asian	Coronary artery disease (CAD)	255/145	120 (47.1%)	46 (31.7%)	7
***GSTP1 null***							
Bhat 2016	India	Mixed	Coronary artery disease (CAD)	200/200	132 (66.0%)	104 (52.0%)	8
Bhatti 2018	India	Mixed	Coronary artery disease (CAD)	560/545	366 (65.4%)	307 (56.3%)	7
Cornelis 2007	Canada	Caucasian	Myocardial infarction (MI)	2042/2042	817 (40.0%)	817 (40.0%)	7
Kariž 2012	Slovenia	Caucasian	Myocardial infarction (MI)	206/257	135 (65.5%)	140 (54.5%)	7
Kovacs 2014	Hungary	Caucasian	Myocardial infarction (MI)	54/78	27 (50.0%)	26 (33.3%)	7
Nomani 2011	Iran	Mixed	Coronary artery disease (CAD)	209/108	118 (56.4%)	60 (55.5%)	8
Phulukdaree 2012	India	Mixed	Coronary artery disease (CAD)	102/100	36 (35.3%)	52 (52.0%)	7
Pourkeramati 2020	Iran	Mixed	Coronary artery disease (CAD)	244/281	64 (26.2%)	56 (19.9%)	8
Ramprasath 2011	India	Mixed	Coronary artery disease (CAD)	290/270	196 (67.6%)	152 (56.3%)	7
Singh 2011	India	Mixed	Myocardial infarction (MI)	230/300	90 (39.1%)	117 (39.0%)	8
Yeh 2013	Taiwan	East Asian	Coronary artery disease (CAD)	458/209	125 (27.3%)	59 (28.2%)	8
***GSTT1 null***							
Abu-Amero 2006	Saudi Arabia	Mixed	Coronary artery disease (CAD)	1054/762	463 (43.9%)	66 (8.7%)	7
Bazo 2011	Brazil	Mixed	Coronary artery disease (CAD)	297/100	69 (23.2%)	19 (19.0%)	7
Bhat 2016	India	Mixed	Coronary artery disease (CAD)	200/200	12 (6.0%)	25 (12.5%)	8
Bhatti 2018	India	Mixed	Coronary artery disease (CAD)	562/564	86 (15.3%)	129 (22.9%)	7
Cora 2013	Turkey	Caucasian	Myocardial infarction (MI)	324/296	106 (32.7%)	63 (21.3%)	8
Cornelis 2007	Canada	Caucasian	Myocardial infarction (MI)	2042/2042	388 (19.0%)	408 (20.0%)	7
Decharatchakul 2020	Thailand	East Asian	Coronary artery disease (CAD)	279/735	115 (41.9%)	242 (32.9%)	8
García 2018	Mexico	Mixed	Coronary artery disease (CAD)	79/101	15 (19.0%)	8 (7.9%)	7
Girisha 2004	India	Mixed	Coronary artery disease (CAD)	197/198	15 (7.6%)	36 (18.2%)	7
Hayek 2006	Israel	Mixed	Coronary artery disease (CAD)	193/2399	30 (15.5%)	392 (16.3%)	8
Kadıoğlu 2016	Turkey	Caucasian	Coronary artery disease (CAD)	29/30	6 (20.7%)	5 (16.7%)	7
Kariž 2012	Slovenia	Caucasian	Myocardial infarction (MI)	206/257	77 (37.4%)	108 (42.0%)	7
Kim 2008	Korea	East Asian	Coronary artery disease (CAD)	356/336	196 (55.0%)	187 (55.7%)	7
Li 2000	USA	Mixed	Coronary artery disease (CAD)	400/890	74 (18.5%)	166 (18.7%)	7
Lakshmi 2012	India	Mixed	Coronary artery disease (CAD)	352/282	81 (23.0%)	39 (13.8%)	7
Levinsson 2014	Sweden	Caucasian	Coronary artery disease (CAD)	112/1221	11 (9.8%)	168 (13.8)	7
Macie 2009	Brazil	Mixed	Coronary artery disease (CAD)	869/1573	209 (24.1%)	337 (21.4%)	7
Manfredi 2007	Italy	Caucasian	Coronary artery disease (CAD)	169/53	95 (56.2%)	13 (24.5%)	7
Manfredi 2009	Italy	Caucasian	Coronary artery disease (CAD)	184/47	84 (45.7%)	13 (27.7%)	7
Martin 2009	USA	Mixed	Coronary artery disease (CAD)	67/63	12 (17.9%)	12 (19.7%)	7
Masetti 2003	Italy	Caucasian	Coronary artery disease (CAD)	308/122	117 (38.0%)	40 (32.8%)	8
Mir 2016	India	Mixed	Coronary artery disease (CAD)	100/100	23 (23.0%)	16 (16.0%)	8
Nomani 2011	Iran	Mixed	Coronary artery disease (CAD)	209/108	16 (7.7%)	17 (15.7%)	8
Norskov 2011	Denmark	Caucasian	Coronary artery disease (CAD)	4930/21684	740 (15.0%)	3161 (14.6%)	7
Olshan 2003	USA	Mixed	Coronary artery disease (CAD)	526/868	75 (14.3%)	165 (19.0%)	8
Palmer 2003	UK	Caucasian	Coronary artery disease (CAD)	51/57	40 (78.4%)	35 (61.4%)	7
Pašalić 2017	Croatia	Caucasian	Coronary artery disease (CAD)	68/177	17 (25.0%)	54 (30.5%)	7
Pourkeramati 2020	Iran	Mixed	Coronary artery disease (CAD)	244/281	129 (52.9%)	143 (50.8%)	8
Ramprasath 2011	India	Mixed	Coronary artery disease (CAD)	290/492	136 (46.9%)	118 (24.0%)	7
Salama 2002	USA	Mixed	Coronary artery disease (CAD)	130/90	32 (26.7%)	14 (15.6%)	7
Singh 2011	India	Mixed	Myocardial infarction (MI)	230/300	23 (10.0%)	61 (20.3%)	8
Tamer 2004	Turkey	Caucasian	Coronary artery disease (CAD)	148/247	48 (32.4%)	70 (28.3%)	7
Tang 2009	China	East Asian	Coronary artery disease (CAD)	277/277	77 (27.8%)	53 (19.1%)	7
Taspinar 2012	Turkey	Caucasian	Coronary artery disease (CAD)	122/142	28 (23.0%)	25 (17.6%)	7
Wang 2008	China	East Asian	Coronary artery disease (CAD)	277/277	77 (27.8%)	53 (19.1%)	8
Wilson 2000	UK	Caucasian	Myocardial infarction (MI)	356/187	90 (25.3%)	36 (19.3%)	8
Wilson 2003	UK	Mixed	Coronary artery disease (CAD)	170/203	34 (20.0%)	44 (21.7%)	7
Yeh 2013	Taiwan	East Asian	Coronary artery disease (CAD)	458/209	276 (60.3%)	110 (52.6%)	8
Zhang 2011	China	East Asian	Coronary artery disease (CAD)	255/145	141 (55.3%)	60 (41.4%)	7

*Abbreviations*: *HWE* Hardy-Weinberg equilibrium, *NOS* Newcastle-Ottawa scale, *NA* Not available

### GSTM1 null polymorphism and the risk of CAD

**Fig. 2:**
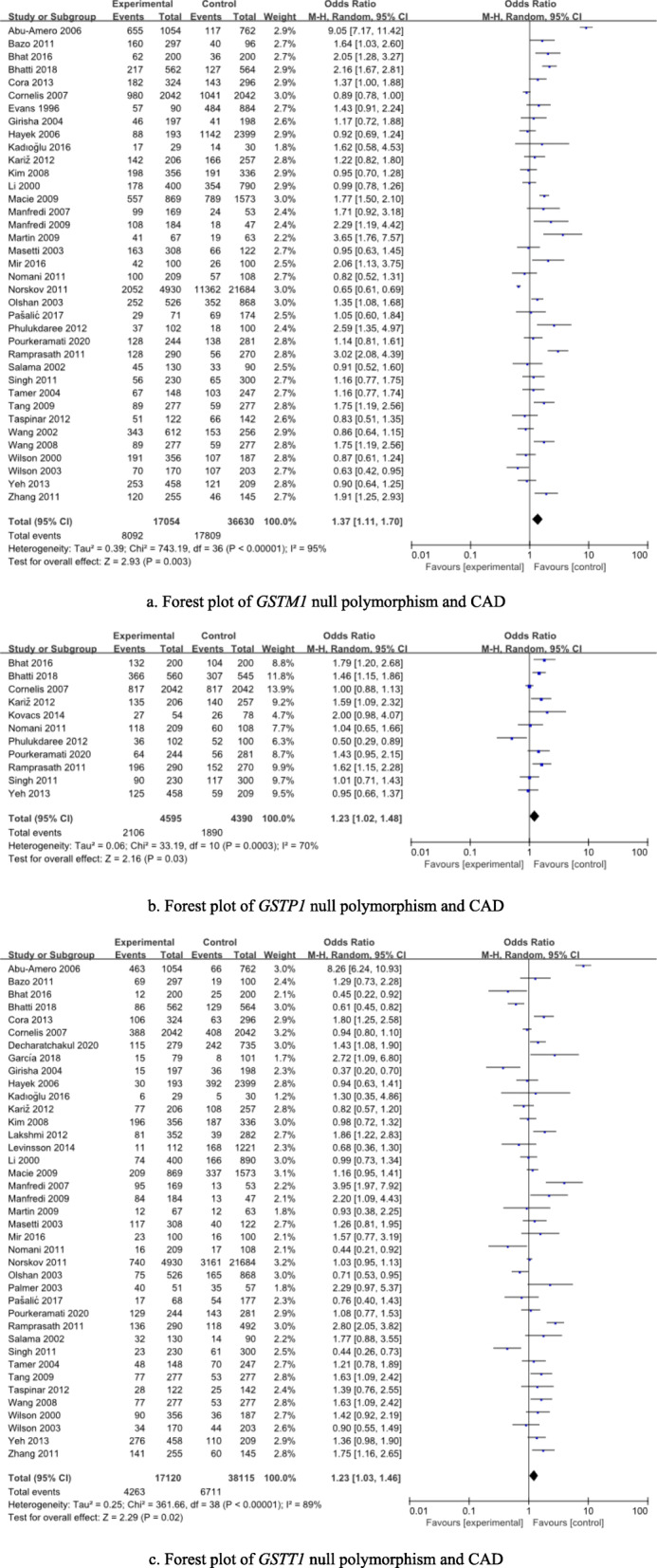
Forest plots for this meta-analysis

Thirty-seven studies (17,054 cases and 36,630 controls) assessed relationship between *GSTM1* null polymorphism and the risk of CAD. The integrated analyses demonstrated that *GSTM1* null polymorphism was significantly associated with the risk of CAD in overall population (OR = 1.37, *p* = 0.003) and mixed population (OR = 1.61, *p* = 0.004) (see Table 2 and Fig. 2).

**Table 2:** Integrated analyses for *GST* null polymorphisms and CAD

Polymorphisms	Population	Sample size (Cases/controls)	Null genotype vs. Present genotype		
			*P* value	OR (95%CI)	I^2^ statistic
***GSTM1 null***	CAD	17,054/36630	**0.003**	**1.37 (1.11-1.70)**	95%
	Caucasian	9501/25537	0.72	1.04 (0.85-1.26)	84%
	East Asian	1623/1244	0.07	1.35 (0.97-1.88)	76%
	Mixed population	5930/9849	**0.004**	**1.61 (1.16-2.22)**	94%
***GSTP1 null***	CAD	4595/4390	**0.03**	**1.23 (1.02-1.48)**	70%
	Caucasian	2302/2377	0.17	1.35 (0.88-2.07)	76%
	Mixed population	1835/1804	0.11	1.23 (0.96-1.59)	68%
***GSTT1 null***	CAD	17,120/38115	**0.02**	**1.23 (1.03-1.46)**	**89%**
	Caucasian	9049/26562	**0.02**	**1.23 (1.03-1.47)**	**67%**
	East Asian	1902/1979	**< 0.0001**	**1.38 (1.20-1.59)**	**36%**
	Mixed population	6169/9574	0.61	1.11 (0.76-1.62)	94%

*Abbreviations*: *OR* Odds ratio, *CI* Confidence interval, *NA* Not available, *CAD* Coronary artery disease

The values in bold represent there is statistically significant differences between cases and controls

### GSTP1 null polymorphism and the risk of CAD

Eleven studies (4595 cases and 4390 controls) assessed relationship between *GSTP1* null polymorphism and the risk of CAD. The integrated analyses demonstrated that *GSTP1* null polymorphism was significantly associated with the risk of CAD in overall population (OR = 1.23, *p* = 0.03) (see Table 2 and Fig. 2).

### GSTT1 null polymorphism and the risk of CAD

Thirty-nine studies (17,120 cases and 38,115 controls) assessed relationship between *GSTT1* null polymorphism and the risk of CAD. The integrated analyses demonstrated that *GSTT1* null polymorphism was significantly associated with the risk of CAD in overall population (OR = 1.23, *p* = 0.02), Caucasians (OR = 1.23, *p* = 0.02) and East Asians (OR = 1.38, *p* < 0.0001) (see Table 2 and Fig. 2).

### Sensitivity analyses

The authors examined stabilities of integrated analyses results by deleting one study each time, and then integrating the results of the rest of studies. The trends of associations were not significantly altered in sensitivity analyses, which indicated that from statistical perspective, our integrated analyses results were reliable and stable (Relevant datasets can be found at https://osf.io, username: suhonglingxxx@163.com, password: suhonglingxxx@).

### Publication biases

**Fig. 3:**
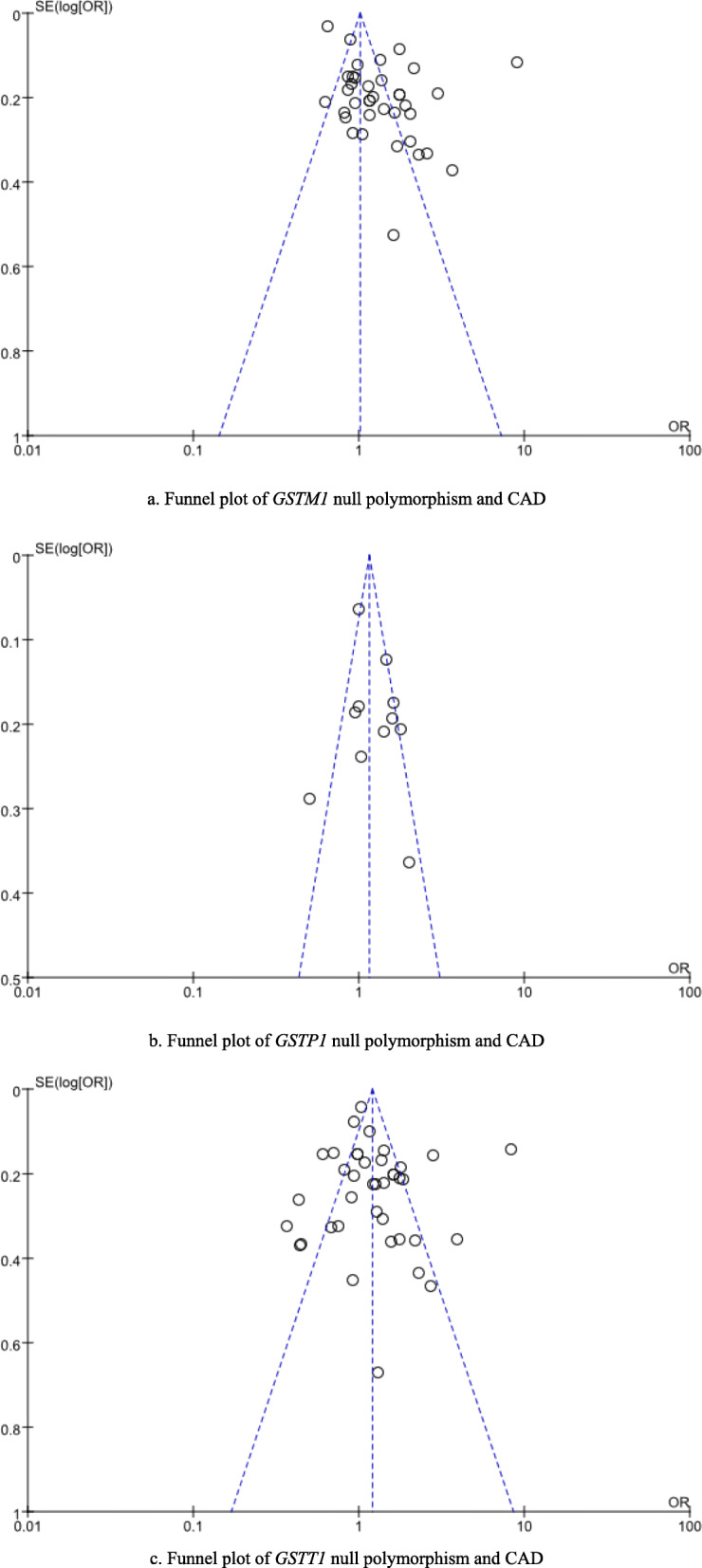
Funnel plots for this meta-analysis

The authors examined potential publication biases in this meta-analysis by assessing symmetry of funnel plots. Funnel plots were found to be generally symmetrical, which indicated that our integrated analyses results were not likely to be severely deteriorated by publication biases (see Fig. 3).

## Discussion

To our knowledge, this is so far the very first meta-analysis regarding associations of *GSTM1* and *GSTP1* null polymorphisms with the risk of CAD, and this is also so far the most complete meta-analysis regarding *GSTT1* null polymorphism and the risk of CAD. The integrated analyses showed that *GSTM1* null, *GSTP1* null and *GSTT1* null polymorphisms were all significantly associated with an increased risk of CAD. Sensitivity analyses suggested that the positive associations observed were quite statistically robust, and no publication bias was detected.

The following points are worth noting when interpreting our integrated findings. Firstly, based on the findings of previous observational studies, we speculated that the investigated *GST* null polymorphisms may lead to a diminished gene expression level of *GST*, which may subsequently affect biological functions of GST, result in excessive oxidative stress and ultimately increase the risk of CAD [11, 12]. Secondly, considering that the functional significances of investigated *GST* null polymorphisms are well established. Our pooled analyses may be still statistically inadequate to detect the actual associations between *GST* null polymorphisms and CAD in certain ethnic subgroups. Therefore, further studies with larger sample sizes in different populations still need to confirm our findings. Thirdly, we want to study all polymorphic loci of the *GST* gene initially. Nevertheless, our comprehensive literature searching did not reveal sufficient eligible studies to support integrated analyses for any other polymorphic loci of the *GST* gene, so we only explored associations with the risk of CAD for three most commonly investigated polymorphisms of the *GST* gene in this meta-analysis. Fourthly, it is worth noting that previously, Song et al. [15] also tried to investigate associations between *GSTT1* null polymorphism and the risk of CAD through a meta-analysis. Nevertheless, this previous meta-analysis only covered relevant genetic association studies that were published before 2014. Since our literature searching revealed that many related studies were published after 2014, an updated meta-analysis like ours is warranted to get more reliable findings. Consistent with the previous meta-analysis, a similar significant finding for *GSTT1* null polymorphism was observed in our integrated analyses. Considering that our updated analyses were derived from more eligible studies, our observations should be considered as a valuable confirmation for pre-existing literatures. Fifthly, *GST* null polymorphisms have also been found to be closely associated with the risk of diabetes, essential hypertension and other types of atherothrombotic disorders such as ischemic stroke or peripheral artery disease [16–20]. Considering that the above mentioned diseases are either considered to be conventional risk factors of CAD or usually manifest as co-morbid conditions of CAD, it would be interesting to perform some stratified analyses accordingly. Nevertheless, due to the fact that the vast majority of eligible studies failed to report genotypic data according to co-morbid conditions, it is impossible for us to conduct such analyses, and we highly recommend future genetic association studies to carry out stratified analyses according to the co-morbid status of these diseases.

The major limitations of our integrated analyses were listed below. Firstly, our integrated analyses results were derived from unadjusted pooling of previous studies. Without access to raw data of eligible studies, we can only assess associations between *GST* null polymorphisms and the risk of CAD based on re-calculations of raw genotypic frequencies provided by eligible studies, and we need to admit that lack of further adjustment for baseline characteristics such as age, gender or co-morbid conditions may possibly influence reliability of our findings [21]. Secondly, environmental factors such as smoking status, eating habits or exercise levels may also influence associations between polymorphisms in *GST* null polymorphisms and the risk of CAD. However, since most of previous studies only paid attention to genetic associations, it is almost impossible for us to explore genetic-environmental interactions in a meta-analysis based on these previous literatures [22]. Thirdly, we did not select ‘grey literatures’ that were not formally published in peer-reviewed scientific journals for integrated analyses because these literatures are generally considered to be incomplete and it is almost impossible for us to extract all necessary data items from these literatures or assess their quality through the NOS scale. Nevertheless, since we did not select ‘grey literatures’ for integrated analyses, despite that funnel plots were found to be overall symmetrical, it should be acknowledged that publication biases still may influence reliability of our integrated analyses results [23].

## Conclusion

In conclusion, this meta-analysis demonstrated that *GSTM1* null, *GSTP1* null and *GSTT1* null polymorphisms were all significantly associated with an increased risk of CAD. These findings suggested that *GSTM1* null, *GSTP1* null and *GSTT1* null polymorphisms may have the potential to serve as genetic biomarkers of CAD and they may be used to identify subjects at higher risk of developing CAD. Further studies with larger sample sizes in different populations are still needed to confirm our findings. Moreover, experimental studies are also warranted to reveal the exact underlying mechanisms of the positive associations observed between above mentioned *GST* null polymorphisms and the risk of CAD in the future.

## Data Availability

Not applicable.

## Abbreviations

*GST*: Glutathione S-transferase
CAD: Coronary artery disease
HWE: Hardy-Weinberg equilibrium
NOS: Newcastle-Ottawa scale
OR: Odds ratios
CI: Confidence intervals
